# Observation of tissues in open aqueous solution by atmospheric scanning electron microscopy: Applicability to intraoperative cancer diagnosis

**DOI:** 10.3892/ijo.2015.2905

**Published:** 2015-02-24

**Authors:** NASSIRHADJY MEMTILY, TOMOKO OKADA, TATSUHIKO EBIHARA, MARI SATO, ATSUSHI KURABAYASHI, MUTSUO FURIHATA, MITSUO SUGA, HIDETOSHI NISHIYAMA, KAZUHIRO MIO, CHIKARA SATO

**Affiliations:** 1 Graduate School of Comprehensive Human Sciences, University of Tsukuba, Tsukuba, Ibaraki 305-0006, Japan; 2 Biomedical Research Institute, National Institute of Advanced Industrial Science and Technology (AIST), Tsukuba, Ibaraki 305-8568, Japan; 3 Traditional Uyghur Medicine Institute of Xinjiang Medical University, Urumqi 830011, Xinjiang Uyghur Autonomous Region, P.R. China; 4 Advanced Technology Division, JEOL Ltd., Akishima, Tokyo 196-8558, Japan; 5 Department of Pathology, Kochi Medical School, University of Kochi, Nankoku, Kochi 783-8505, Japan

**Keywords:** ASEM, tissue, heavy metal staining, scanning electron microscope, ClairScope, kidney, metastatic murine breast cancer

## Abstract

In the atmospheric scanning electron microscope (ASEM), a 2- to 3-μm layer of the sample resting on a silicon nitride-film window in the base of an open sample dish is imaged, in liquid, at atmospheric pressure, from below by an inverted SEM. Thus, the time-consuming pretreatments generally required for biological samples to withstand the vacuum of a standard electron microscope are avoided. In the present study, various mouse tissues (brain, spinal cord, muscle, heart, lung, liver, kidney, spleen and stomach) were fixed, stained with heavy metals, and visualized in radical scavenger D-glucose solution using the ASEM. While some stains made the nuclei of cells very prominent (platinum-blue, phosphotungstic acid), others also emphasized cell organelles and membranous structures (uranium acetate or the NCMIR method). Notably, symbiotic bacteria were sometimes observed on stomach mucosa. Furthermore, kidney tissue could be stained and successfully imaged in <30 min. Lung and spinal cord tissue from normal mice and mice metastasized with breast cancer cells was also examined. Cancer cells present in lung alveoli and in parts of the spine tissue clearly had larger nuclei than normal cells. The results indicate that the ASEM has the potential to accelerate intraoperative cancer diagnosis, the diagnosis of kidney diseases and pathogen detection. Importantly, in the course of the present study it was possible to increase the observable tissue area by using a new multi-windowed ASEM sample dish and sliding the tissue across its eight windows.

## Introduction

Optical microscopy (OM) of tissues has developed in various ways leading to great discoveries and advances in biology and medicine (1,2). Tissue samples are either prepared by time-consuming paraffin embedding at room temperature (RT) and cut, or quickly frozen and cut at cryo-temperatures. Paraffin-embedding is widely used in basic biological studies, applied biology fields and medicine. In the quick cryo-sectioning method, excised tissues are frozen in OCT compound, cryo-sectioned at a thickness of 3–5 μm, fixed, stained by hematoxylin-eosin (H&E) and inspected during surgery performed to remove tissue suffering from cancer invasion and metastasis (3,4). The size of cell nuclei is the most significant indicator used for the intraoperative cancer diagnosis, the nucleus of cancer cells generally being larger than that of normal cells. However, cryo-thin-sectioning is quite difficult and takes ~15–30 min for each sample, thus, the procedure can prolong the whole operation, which is a burden for patients.

**Figure 1:**
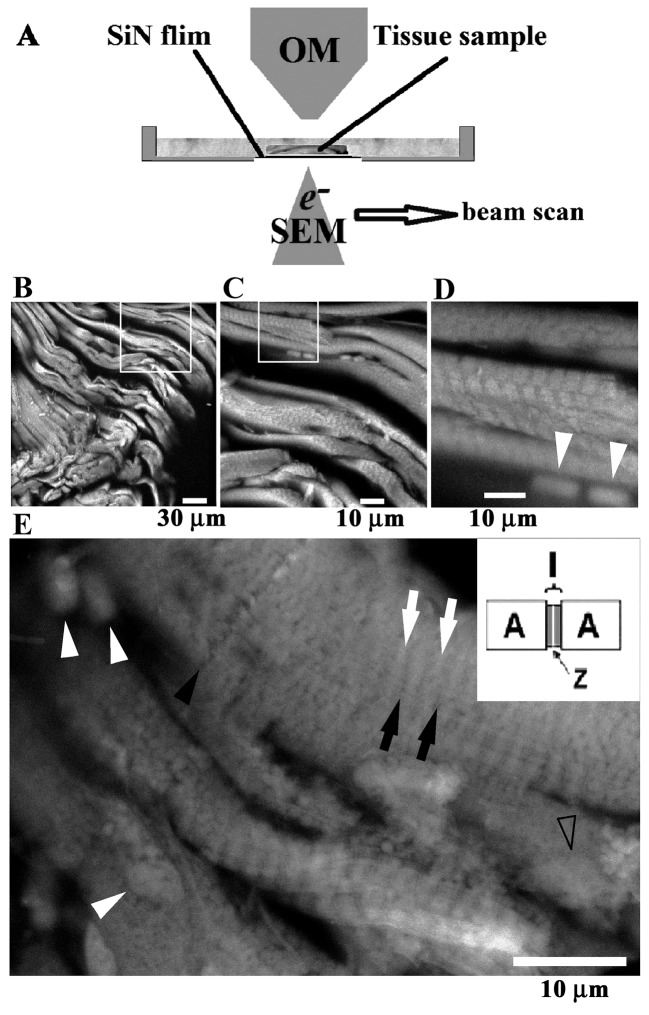
Schematic diagram of the ASEM and observation of heart tissues. (A) In the ASEM correlative microscope, tissue can be observed in aqueous liquid by the SEM from below through the SiN film of the ASEM dish and by OM from above, allowing various manipulations. Since the optical axes of the two microscopes are aligned, the central region of the sample overview obtained by OM can be imaged immediately afterwards at higher resolution by SEM. (B) Slab of heart tissue stained with PTA, immersed in radical scavenger D-glucose and observed by ASEM at low magnification. Longitudinal cardiomyocytes are evident and sometimes branched. (C and D) Higher magnification image of the white rectangle in the preceding panel. Myofibrils can be brightly distinguished above the background. (E) ASEM image of another cardiac muscle area. Nuclei (open arrowhead) and intercalated discs (black arrowhead) are visible; the nuclei between myocytes (white arrowheads) might belong to attached fibroblasts. Sarcomere striations are visible in myocytes: A-bands (white arrow) are broad bright zones, while I-bands are dark zones (black arrow). The Z-line can be faintly recognized as an indistinct thin white line at the center of I-bands. Inset, diagram of the sarcomeres.

The surface level of tissue blocks excised from various organs (heart and kidney) (5), kidney (6), brain tumor (7,8), liver, sciatic nerve, spinal cord (9), colon (10) has been successfully observed in an environmental capsule by scanning electron microscopy (SEM). Environmental capsules allow ‘wet’ samples to be examined in the vacuum of the microscope, avoiding dehydration artifacts. However, the samples are not accessible for additional staining or manipulation. The Atmospheric SEM (ASEM) ClairScope™ was developed to realize SEM of a sample at atmospheric pressure in a readily-accessible, open container (ASEM dish) (11). The microscope has an inverted SEM configuration, the column being closed by the ASEM dish at the top. An optical microscope (OM) positioned above the ASEM dish can be used to observe wide areas of the sample, and the SEM to image specified smaller regions through a thin SiN film in the base of the dish (Fig. 1A). The optical axes of both microscopes are aligned and fixed to ensure that correlative images are recorded, and the specimen stage can move two-dimensionally (x–y) for targeting. The specimen depth observable by ASEM is 2–3 μm at 30 kV (12,13) and the resolution is 8 nm near the SiN membrane, which is advantageous for the observation of fine subcellular structures. The detachable 35-mm ASEM dish allows the culture of various primary cells and subsequent efficient staining-washing cycles (12,14–16). We have cultured primary cells sampled from various mammalian (12,14) and insect organs (14,15), including mouse hippocampus (12), cortex and cerebellum (14), and from prokaryotes, including coccus, bacillus (15) and mycoplasma (17). Cultured cells were successfully stained with different heavy metals and imaged (11). Because the epitopes of the cells were preserved in the aqueous solution, we were also able to successfully immunolabel different cells with various antibodies, including mouse monoclonal antibodies (12,14,15,17–20). Furthermore, we recently used ASEM to observe TI-Blue-stained tissue from a gold fish brain, as briefly outlined in the study of Nishiyama *et al* (11) (Fig. 9).

In the present study, we report the observation of various mouse tissues by ASEM after staining with heavy metals, and indicate the possible use of ASEM for the diagnosis of cancer and kidney diseases. Staining with phosphotungstic acid (PTA) or platinum blue (Pt-blue) made cell nuclei very prominent, while staining with uranyl acetate (UA) or by a modified NCMIR method clearly revealed cell outlines, organelles and extracellular structures. The difference between normal and cancer-metastasized tissue was successfully visualized for lung and spinal cord specimens. Importantly, the area observable by ASEM was enlarged both by the use of a new multi-windowed sample dish and by exploiting the open ASEM dish configuration to slide tissue across the windows.

## Materials and methods

### Animals

Six to fifteen week-old male and female ICR mice and 8–12-week-old female BALB/c mice (Japan Clea, Tokyo, Japan) were sacrificed to obtain normal tissue for observation. Tumor bearing mice were obtained 10 days after the intravenous injection of 4T1E/M3 breast cancer cells (1×10^6^/mouse) or 30 days after the subcutaneous injection of the cancer cells (1×10^6^/mouse) to 8-week-old female BALB/c mice (21–23). The animal studies were in compliance with the national institute rules of conduct and adhered to the principles of Institutional Animal Care and Use Committee Guidebook. All experiments were approved by the Animal Care and Use Committee of the National Institute of Advanced Industrial Science and Technology (AIST).

### Tissue sample preparation

Animals were anesthetized using isoflurane (Abbott, Maidenhead, UK) and sacrificed by intracardiac perfusion of 4% paraformaldehyde (PFA; Wako Pure Chemicals, Osaka, Japan) in phosphate-buffered saline (PBS, pH 7.4) (24). Tissues were either cut with a scalpel to obtain 1–2-mm thick tissue slabs, or sliced with a PRO7 linear slicer (Dosaka, Kyoto, Japan) to obtain 100–200 μm thick slabs. Samples were washed several times in PBS, and further fixed with 4% PFA or 1% glutaraldehyde (GA) (Nisshin EM, Tokyo, Japan) or with a solution containing both fixatives, for 30 min at RT; the fixative volume was 15–20 times the sample volume.

### Histology for OM

Lungs and spines harvested from tumor bearing mice were fixed in 10% neutral buffered formalin (Wako Pure Chemicals) at RT. The spines were then decalcified in decalcifying solution A (Wako Pure Chemicals) following the manufacturer’s protocol. Fixed lungs, spleens or decalcified fixed spines were then embedded in paraffin, cut into 5–6 μm sections, stained with H&E and inspected and photographed by OM.

### Heavy metal staining

PTA or Pt-blue (TI-Blue; Nisshin EM), 6% solution; Pt_4_(NH_3_)_8_(C_6_H_13_O_5_)_4_) staining: the fixed tissues were perforated with 0.5% Triton X-100 (MP Biomedicals, LLC, Illkirch, France) in PBS at RT for 15 min, and washed with DDW twice. They were stained with 2% PTA (TAAB Laboratories Equipment Ltd., Aldermaston, UK) or 0.6% TI-Blue or double stained with both, for 3 h at RT or overnight at 4°C.

UA staining: the tissues were incubated in filtered 1% tannic acid (Polysciences Inc., Warrington, FL, USA), 1% GA for 30 min at RT, and washed with DDW twice. They were stained with filtered 2% uranyl acetate (UA) for 30 min at RT.

Quick method: without prior perforation, fixed tissues were incubated with 2% UA for 15 min at RT.

NCMIR staining method: tissues were stained using a slight modification of the NCMIR method developed by the Ellisman group (25). The fixed tissues were perforated with 0.5% Triton X-100 in PBS at RT for 15 min, washed with DDW, and further fixed with 2.5% GA, 2% PFA in filtered 0.1 M phosphate buffer (PB, pH 7.4) containing 2 mM CaCl_2_ at RT for 15 min. They were then washed using filtered 0.15 M PB containing 2 mM CaCl_2_, and further fixed/stained with the same buffer supplemented with 1.5% potassium ferricyanide (Sigma-Aldrich, St. Louis, MO, USA), 2% aqueous osmium tetroxide (OsO_4_) (Nisshin EM) at RT for 60 min. After washing with DDW, tissues were then incubated with filtered 1% thiocarbohydrazide (TCH; Tokyo Chemical Industry, Co., Ltd., Tokyo, Japan) (25) at RT for 20 min, rinsed with DDW, further stained with 2% aqueous OsO_4_ at RT for 30 min, rinsed with DDW, stained with 2% UA in DDW and kept at 4°C overnight. Finally, after rinsing with DDW, the tissue samples were stained with 0.4% lead citrate (TAAB Laboratories Equipment) at RT for 2 min.

Once fully stained, all samples were placed ‘cut’ face down in the ASEM dish immersed in radical scavenger solution and imaged as soon as possible by OM and SEM, as the contrast of the most stains fades on storage.

### ASEM imaging

The ClairScope ASEM system (JASM-6200; JEOL Ltd., Tokyo, Japan) was used to record SEM images (11). Unless otherwise stated, the standard 35-mm ASEM dish was used; the 100-nm thick (250×250)-μm SiN film window built into its base separates the sample immersed in aqueous liquid from the vacuum inside the SEM column. A newly-developed dish, with eight 100-nm thick (250×250)-μm SiN film windows was employed to observe larger areas. After fixation and staining, tissues were placed on the SiN film window, and observed in 10 mg/ml (w/v) D-glucose (Dextrose; MP Biomedicals LLC) or ascorbic acid (L-Ascorbic acid; Sigma-Aldrich) in DDW, using the inverted SEM of the ASEM. When the uneven surface of tissue prevented it from attaching well to the film, 150–250 Pascal pressure was applied during imaging. To achieve this a cover glass was positioned on the upper surface of the tissue and loaded with a lead weight. The acceleration voltage of the SEM was 30 kV, electrons backscattered from the cells were recorded for visualization. As the stain scatters more electrons than tissue, more heavily stained regions are bright in the ASEM images.

### Sliding a stained tissue slab across the ASEM dish

A stained tissue slab was placed on the multi-windowed ASEM dish and imaged in 10 mg/ml (w/v) D-glucose or ascorbic acid in DDW from underneath by the inverted SEM. The tissue was then pushed slightly to the side with tweezers under monitoring by OM from above. The tissue was again imaged by SEM, and the images before and after the movement were merged.

## Results

As detailed below, ASEM was successfully employed to image a range of mouse tissue samples*.* In each case tissue slabs were stained with heavy metal solution, laid cut face down on the SiN film of the ASEM dish, and observed in liquid by ASEM (see Materials and methods).

### Cardiac muscles

Hearts of mice sacrificed by intracardiac perfusion of 4% PFA in PBS were dissected into 1- to 2-mm slabs, and either further fixed with PFA alone or doubly with PFA and GA in PBS. After perforation with Triton X-100, each tissue slab was stained with PTA and imaged by ASEM (Fig. 1A). Cardiomyocytes were evident (Fig. 1B–E) and their more highly stained nuclei were observed. The nuclei of what are presumably surrounding cells, possibly attached fibroblasts, were observed as blurred white ellipses (Fig. 1D and E). This blurring is probably attributable to the distant location of the nuclei from the supporting SiN film, as demonstrated earlier for gold particles (11,15). Intercalated discs could be clearly distinguished. The alternating dark and bright bands imaged by ASEM can be interpreted as thin dark zones (I-bands) and broad bright zones (A-bands) of myofibril sarcomeres. Z-lines were also faintly imaged, each appearing as a thin white line in the center of a dark I-band.

### Neural tissues

**Figure 2:**
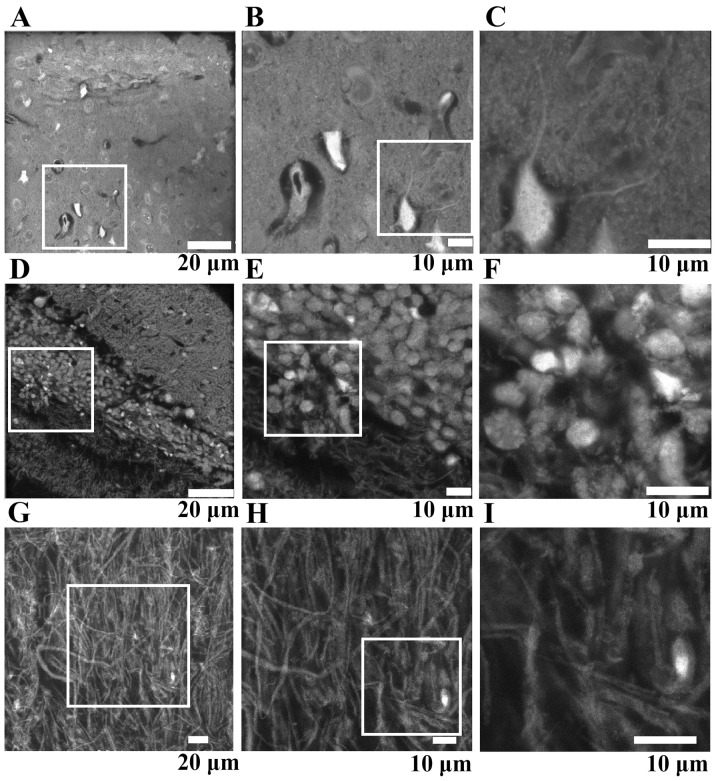
ASEM images of neural tissues stained with PTA. In these images, bright nuclei are distinguished above the background, while cytoplasm and nerve fibers are darker, as they were only weakly stained. (A) Vertical section of the cerebrum showing a layered structure. (B and C) Higher magnification image of the white rectangle in the preceding panel. Different kinds of neurons with nuclei have various dendrites and are surrounded by glia cells. (D) Horizontal section of the cerebellum. The three clearly different layers visible are probably the molecular layer, the granular layer and cerebellar white matter. (E and F) Higher magnification images. Nuclei have brightly stained patches that are probably chromatin and nucleoli. (G) Vertical section of thoracic spinal cord. Neural networks can be distinguished, mainly running from top to bottom. (H and I) Higher magnification images. Minor connections in various directions are apparent.

Nerve tissues from ICR mice were stained with PTA and similarly imaged by ASEM. Observation of the surface level of the tissues showed that neurons extend their nerve fibers to form fine networks surrounded by glia cells (Fig. 2). Imaging the cerebrum revealed a highly ordered structure of cells and a systematic network of neurons including delicate connections (Fig. 2A–C). In the cerebellum, three clearly different layers were observed (Fig. 2D), presumably the molecular layer, the granular layer and cerebral white matter. Again, neurons were connected by delicate systematic networks formed by nerve fibers (Fig. 2E and F). At higher magnification, nuclei were seen to include brightly stained patches that were presumably chromatin and nucleoli (Fig. 2F).

The spinal cord contains a larger number of fibrous structures than the brain. Imaging vertical sections of spinal cord by ASEM revealed 200 nm - 3 μm thick, almost perpendicular filament ‘strings’ together with a lower number of filaments running in various directions, overall forming a complex network. Bright nuclei were sometimes observed at the end of the strings (Fig. 2G–I).

### Liver

**Figure 3:**
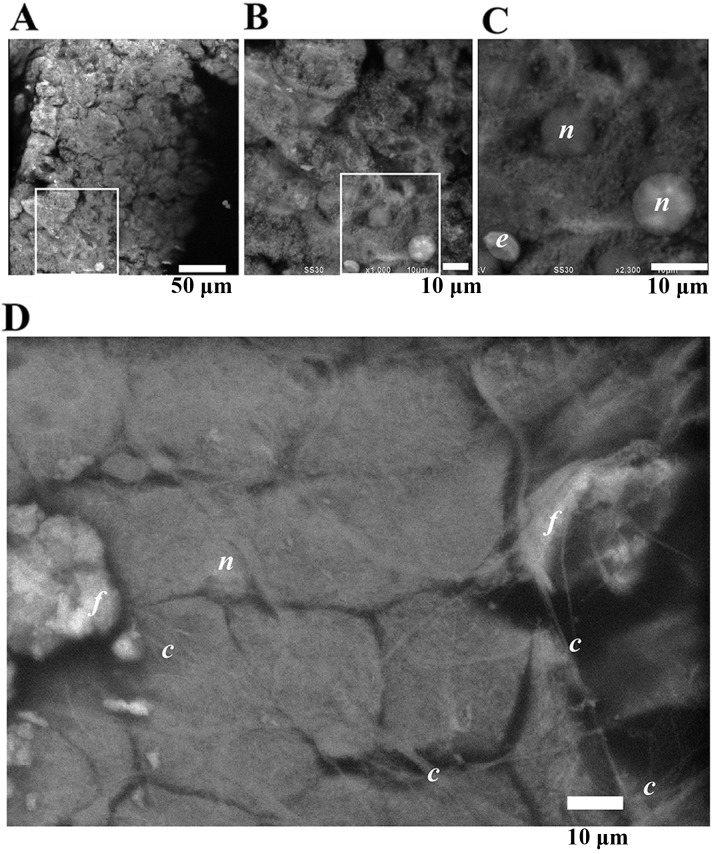
ASEM images of liver. (A) Low magnification image of a tissue slab stained with UA. Hepatocytes, and probably sinusoids are visible. (B and C) Higher magnification image of the white rectangle in the preceding panel. Hepatocytes, sometimes with their nucleus (n) near the SiN support, can be clearly distinguished from erythrocytes (e). The brightly stained patches evident within some nuclei are probably chromatin and nucleoli. (D) Hepatocytes at the surface of a different tissue slab stained with PTA. Slightly more heavily stained collagen-like fibers (c) are visible as brighter strands. A nucleus (n) and fat droplet-like structures (f) can also be observed.

When liver tissue slabs were stained with UA, hepatocytes, erythrocytes and probably sinusoids in the surface layers were visualized by ASEM (Fig. 3A–C). The nuclei of hepatocytes were also sometimes distinguishable (Fig. 3C). When other tissue slabs were stained with PTA and imaged, collagen fibers and fat droplet-like structures were also prominent (Fig. 3D). However, it is not easy to evaluate the staining preferences of UA and PTA using different samples.

### Kidney

**Figure 4:**
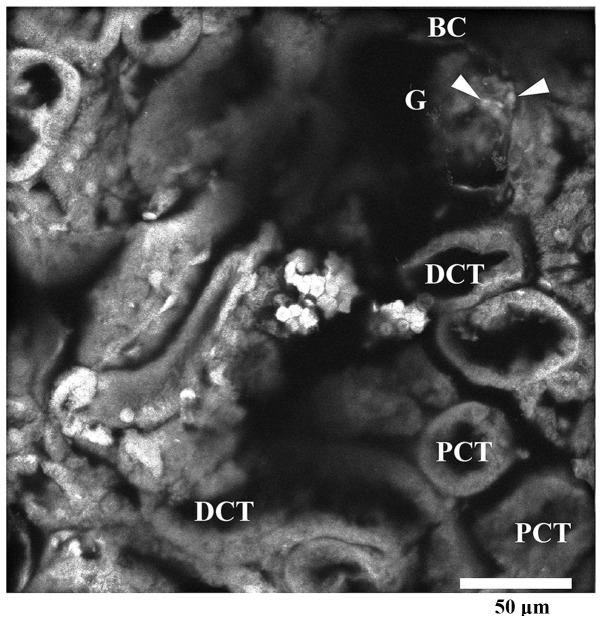
ASEM image of kidney cortex tissue stained with PTA. The features evident in the characteristic view are putatively assigned as: Bowman’s capsule (BC), podocytes (visceral epithelial cells; white arrowheads), a glomerulus (G), proximal convoluted tubules (PCT) and distal convoluted tubules (DCT).

A slab excised from a mouse kidney was imaged by ASEM without any pretreatment other than fixation, permeation and PTA staining (Fig. 4). Bowman’s capsules, glomeruluses, proximal or distal convoluted tubules and also podocytes were visualized (Fig. 4). The overviews are consistent with images obtained when traditional Epon-thin sections of kidney biopsies are inspected by transmission electron microscopy (TEM). Importantly, sample preparation took much less time, indicating the potential of the ASEM method for quick diagnosis or to rapidly obtain a second opinion. Accelerating the sample preparation further by omitting the perforation step (see Materials and methods; quick method), delivered images of basically the same quality (data not shown) as those obtained by the standard ASEM staining protocol including perforation.

### Skeletal muscle

**Figure 5:**
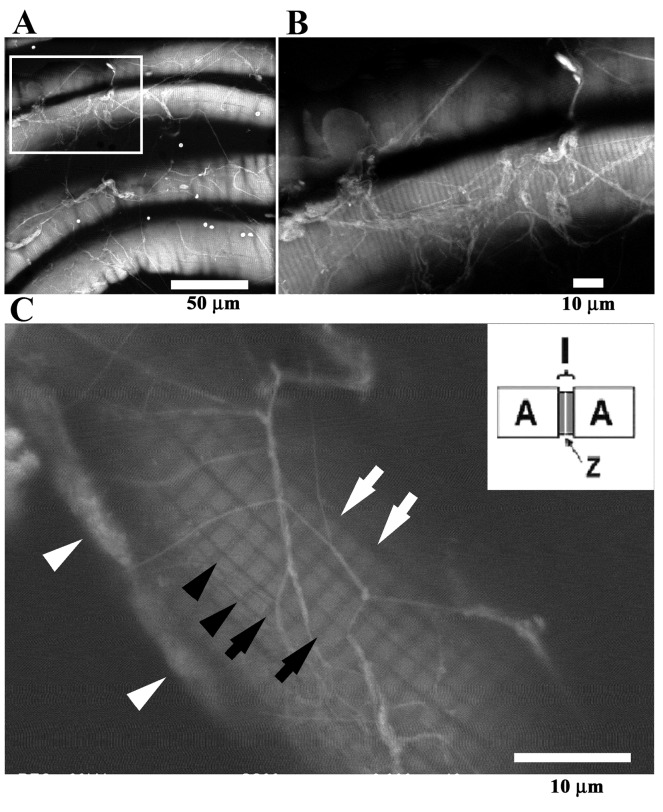
ASEM images of gastrocnemius skeletal muscle stained with PTA. (A) Low magnification image. (B) Higher magnification image of the white rectangle in the preceding panel. Filament networks are evident as bright strands on striped striated muscle fibers. (C) Another specimen area. The muscle fiber has A-bands, evident as broad bright zones (white arrows), and I-bands, evident as dark zones (black arrows). Z-lines look like a faint thin white line in the middle of the I-bands (black arrowheads). Nuclei are bright (white arrowheads). Inset, diagram of the sarcomere.

Muscle fibers were clearly visible when PTA-stained tissue slabs of gastrocnemius muscle were observed by ASEM (Fig. 5). The myocytes were found to be surrounded by filaments (Fig. 5A and B). In myocytes, I-bands and A-bands were visualized as dark zones and broader bright zones, respectively, and Z-lines were frequently distinguished as a fine thin line in the center of dark I-bands (Fig. 5C). These results suggest that myocytes can be easily imaged at high resolution using the ASEM.

### Stomach

**Figure 6:**
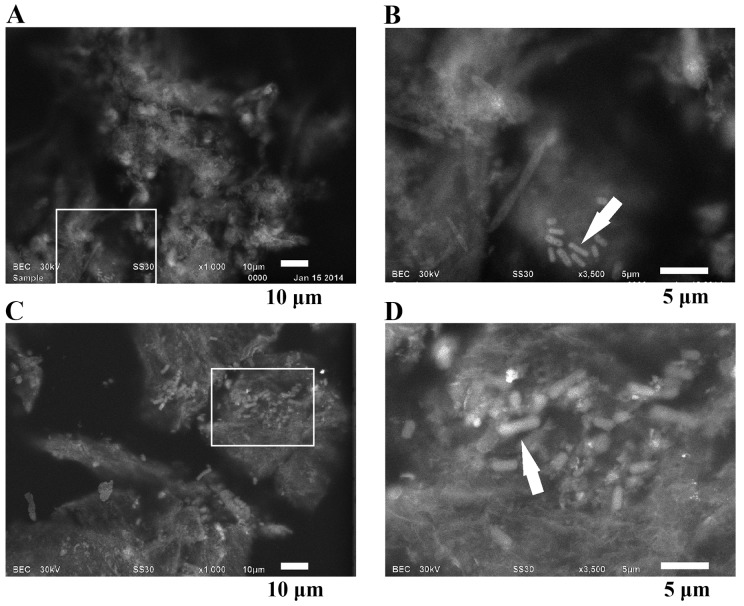
ASEM images of digestive tract. (A) Low magnification images of the mucosal side of stomach stained with PTA. (B) Higher magnification image. Commensalism of bacteria (arrow) is revealed. (C) Another area of stomach stained by the modified NCMIR method. (D) Higher magnification image; bright symbiotic bacteria (arrow) are observed.

Mucosa of the stomach lumen were observed together with symbiotic bacteria (Fig. 6A and B); a bacteria colony can be seen in Fig. 6B (arrow). Staining by the modified NCMIR method instead of with PTA, delivered clear images of the symbiotic bacteria revealing their different shapes (Fig. 6C and D).

### Spleen

**Figure 7:**
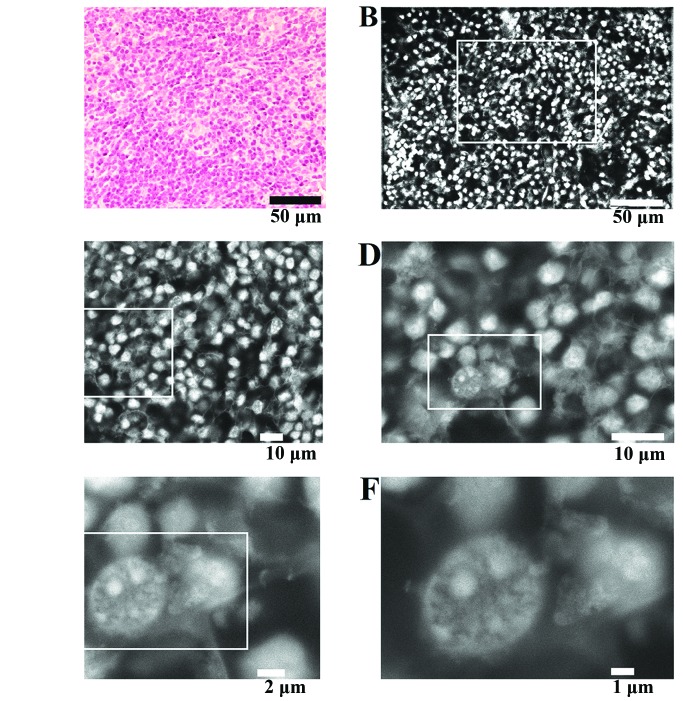
Images of spleen tissue. (A) OM. The spleen was cross-sectioned to obtain 5- to 6-μm, thin-sections, H&E stained and observed by OM. (B) ASEM. The spleen was independently cross-sectioned to obtain 200-μm-thick tissue slabs, stained with PTA and observed at low magnification by ASEM. (C–F) Higher magnification ASEM images of the white rectangle in the preceding panel. Differently shaped blood cells, presumably including lymphocytes, were observed.

H&E-stained thin-slices of spleen were observed by OM, revealing a large number of round cells with and without filopodia, presumably hematopoietic cells (Fig. 7A). The surface layers of independently cut, thicker slabs of spleen tissue stained with PTA looked similar when imaged at low magnification by ASEM (Fig. 7B). In a typical area there were many differently shaped cells. From their shapes and nuclei, some of them were probably lymphocytes, e.g., B cells, T cells or plasma cells (Fig. 7C–F). At higher magnification, nuclei were found to have brightly stained patches, presumably nucleoli and chromatin (Fig. 7F compared to Figs. 2F and 3C). Fibrous structures <2 μm in thick, were also observed. These results suggest that ASEM can be employed to study lymph cells, including their development and immunity in the spleen.

### Comparative observation of normal lung and the lung metastasized by breast cancer

**Figure 8:**
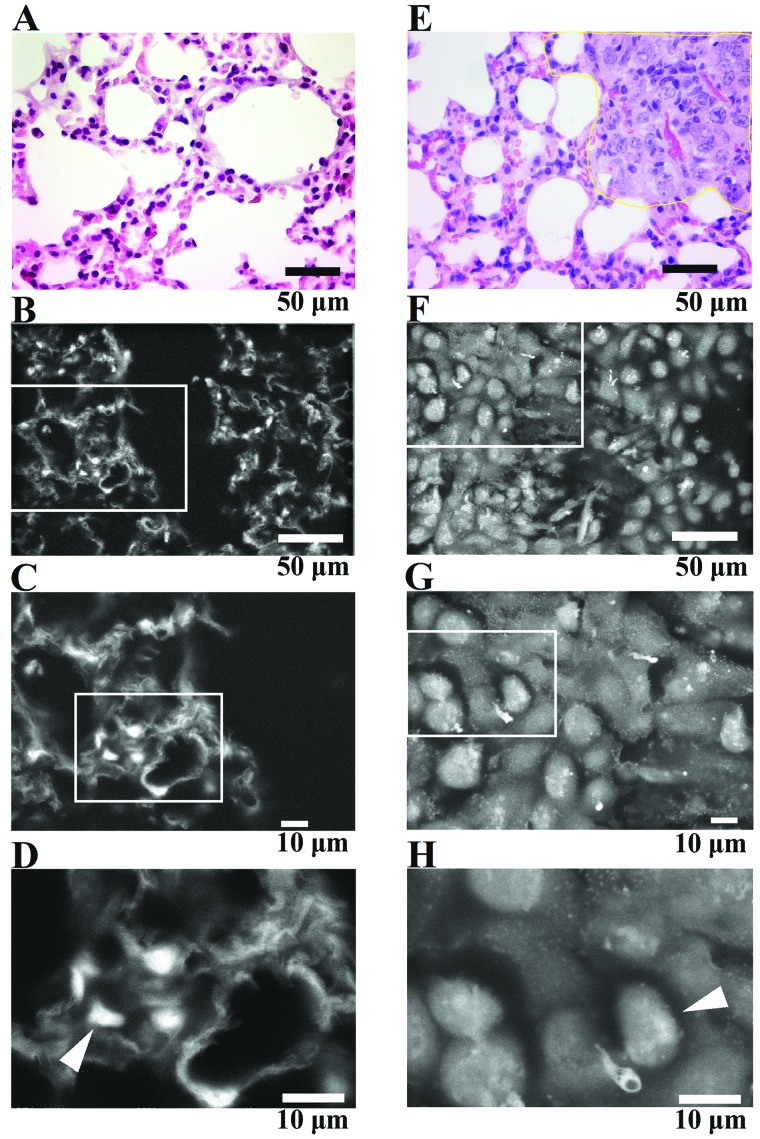
Comparative ASEM observation of normal lung and lung metastasized by breast cancer cells. (A) OM of an H&E-stained thin-section of normal lung. Nuclei are stained blue and the cytoplasm is stained red. (B) Low magnification ASEM image of an independently-prepared tissue slab stained with both Pt-blue and PTA. Alveoli with alveolar ducts, a vein system and trachea are visible. (C and D) Higher magnification images of the white rectangle in the upper panel. Normal size nuclei are observed (arrowhead). (E–H) Comparative observation of tissue excised from a lung metastasized with breast cancer cells. (E) OM of an H&E-stained thin section. (F–H) ASEM of an independently-prepared Pt-blue- and PTA-stained thick slab at the same magnification as the corresponding left panels. Regular alveoli, alveolar ducts and alveolar cells are only faintly discernable. Most of the space is occupied by cells of different shapes with larger nuclei (arrowhead), i.e., cancer cells.

The lungs are known to be organs easily metastasized by cancer. Lung tissues excised from normal mice and from mice with tumors induced by the injection of breast cancer cells, were stained with both Pt-blue and PTA and observed by ASEM. OM of H&E-stained thin lung sections from other normal (Fig. 8A) and metastasized mice (Fig. 8E) was independently performed for comparison. Observed by ASEM, the normal lung tissue showed typical thin-wall structures with alveoli, alveolar ducts, a vein system and trachea (Fig. 8B–D). In the cells, nuclei close to the surface of the tissue slabs were observed as bright features in the images. Their outlines were clear, facilitating the measurement of their diameters. By contrast, only faint traces of the regular alveoli and alveolar ducts were discernable for metastasized lung (Fig. 8F–H); most of the alveolar system had been replaced by differently shaped cells with larger nuclei, i.e., breast cancer cells (Fig. 8H). These results are consistent with those obtained by H&E staining and OM (Fig. 8A and E), suggesting that ASEM could be used for cancer diagnosis.

### Normal spinal cord and the spinal cord metastasized by breast cancer

**Figure 9:**
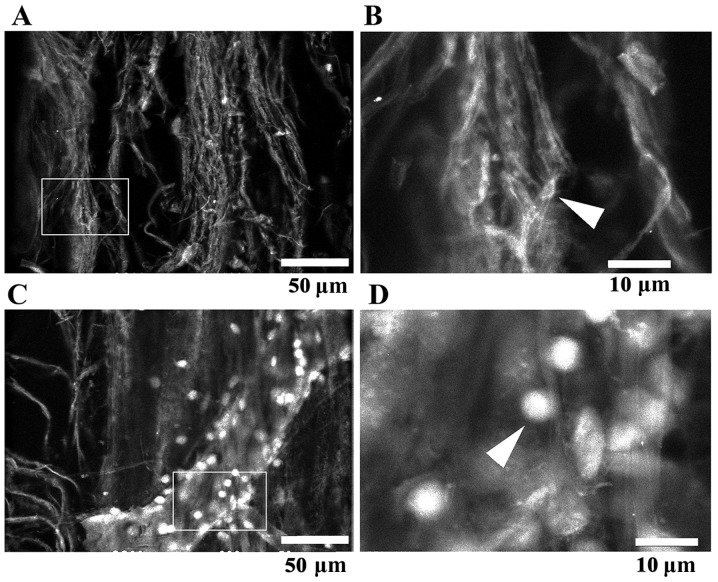
Comparative ASEM observation of normal spinal cord and spinal cord metastasized by breast cancer cells. Tissues are stained with both Pt-blue and PTA. (A and B) ASEM image of a slab of normal spinal cord. Normal size nuclei are observed at the end of fibers (arrowhead). (C and D) Comparative observation of tissue excised from a spinal cord metastasized with breast cancer cells. Unusually large nuclei similar to those seen in lung metastasized by breast cancer cells (Fig. 8F–H), were observed (arrowhead).

Spinal cord excised from the mice with tumors induced by the injection of breast cancer cells, were stained with both Pt-blue and PTA and compared with those from the normal mice using the ASEM. While nuclei seen at the end of the fibers in normal spinal cord were all the correct size (Fig. 9A and B, arrowhead), a thin film-like tissue of cells with unusually large nuclei was observed in a metastasized spinal cord (Fig. 9C and D, arrowhead). The large nuclei were similar in size to those seen in lung metastasized with breast cancer cells (compare Fig. 8E–H).

### Wide area observation by sliding tissue on the ASEM dish

**Figure 10:**
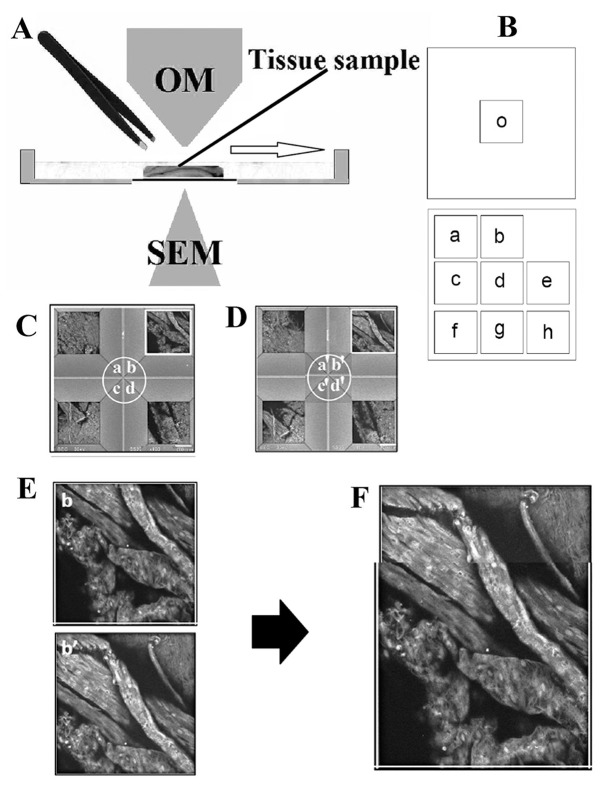
Wide area imaging by shifting a tissue on the ASEM dish. (A) Schematic diagram of the procedure. The shift caused by pushing with the tweezers is precisely controlled by low magnification monitoring using the upper OM (see Fig. 1A). (B) Schematic diagrams of a one-window and an 8-window ASEM dish. All windows are 250×250 μm. (C) Images of spinal cord tissue initially recorded from windows a, b, c and d. (D) Images recorded in the same windows after the tissue has been pushed in one direction causing it to slip across the SiN-film window. (E) Higher magnification image of window b in panel C and of window b′ in panel D. (F) Merged image of windows b and b′.

To enlarge the area observable by ASEM, a spinal cord slab stained with PTA was placed in a newly developed 8-window ASEM dish and the tissue was repeatedly induced to slide very slightly across the thin membrane windows and imaged (Fig. 10A). Although the observable area in each ASEM imaging step was restricted to the field of the 8 windows, two sequential images partly overlapped and could be merged, covering a wider area of the spinal cord (Fig. 10C–F). The observable area of the multi-windowed dish (Fig. 10B) was thus successfully extended. The shift of the tissue on the flat base of the dish (Fig. 10A) was precisely monitored by OM from above. This was made possible by the open ASEM dish configuration and the axis-aligned OM (Fig. 1A). In the future, the procedure could be automated by constructing a micro motor-driven manipulator to realize an accurate shift at regular time intervals synchronized with ASEM scanning.

## Discussion

SEM has proved very important to the study of morphology, especially the surface morphology of tissues. To prevent deformation artifacts, tissue samples generally require time-consuming pretreatment, including dehydration and metal coating, before they are placed in the vacuum of the microscope. Dehydration, metal coating and the other tedious sample pretreatments are not required for ASEM.

ASEM is a unique imaging technique with many special characteristics. First, samples can be observed in liquid at atmospheric pressure. This is made possible by the use of an open dish-shaped sample holder and an inverted SEM. The former seals the top of the SEM column and has a thin SiN-film window in its base, allowing SEM images of the sample to be obtained from below. Second, an OM positioned above the open ASEM dish facilitates the outside manipulation of the sample during observation. Third, the electron-conducting aqueous solution employed eliminates charging of the sample. Fourth, the detection of backscattered electrons allows a 2- to 3-μm slice of the specimen resting on the flat SiN-film window to be observed when the SEM is operated at an accelerating voltage of at 30 kV (12,13,26). This means that diced and stained tissues can be viewed quickly without further thin-sectioning, providing structural information about the connectivity and organization of cells and the fine intracellular and extracellular features near the SiN film.

The oxidized surface of the SiN film, SiO_x_, formed during the fabrication process is considered to have a glass-like nature. Certain cell types, e.g., HEK, COS, HeLa and B16 melanoma cells, can grow directly on this film and on the glass base of the sample dish. However, the culture of primary cells directly isolated from mouse tissues is facilitated if the surfaces are first coated by poly-L-lysine or proteins (12,14,15). As a hippocampus slice from mammal brain has been successfully cultured on cover glass, organotypic cultures (27,28) are highly expected to grow on the ASEM dish, allowing their study at high resolution. Because the dish accommodates 2 ml of liquid, it provides a stable culture environment for organs. It eliminates the delicate handling of a few tens of microliters of liquid containing a tiny piece of tissue, required to charge an environmental capsule. Furthermore, it facilitates the administration of drugs (29), making drug screening a further possible application of the ASEM. The open configuration of the ASEM dish also enables micro-injection or electrophysiology of tissues, monitored by OM.

In the present study, the staining reagents UA, Pt-blue, PTA, OsO_4_ and lead citrate, were successfully employed for tissue observation by ASEM. In particular, the bright images of chromatin and nucleoli observed in nuclei stained with Pt-blue and/or PTA (Figs. 2F, 3C and 7F) suggest that these moderate and economical reagents can be used to visualize nucleotide-protein complexes. This, together with earlier images of chromosomes (11), raises the possibility of applying Pt-blue/PTA staining and ASEM to chromatin-related fields, e.g., spermatology. High resolution karyotyping using ASEM might help to find slight chromosomal deviations that are difficult to observe at present.

Simple PTA staining and ASEM visualized a bacteria colony on the mucosal side of stomach tissue (Fig. 6). If the staining time can be reduced, ASEM has the potential to visualize the characteristics of bacteria without further culturing, which would be especially important for infectious bacteria that are viable but non-culturable (VNC bacteria), and for anaerobes that take a relatively long time (several days) to culture for diagnosis. Furthermore, ASEM could be applied to visualize the infiltration of bacteria into tissue regions and the inflammation they trigger during surgery and treatment. The modified NCMIR staining method involving the use of OsO_4_ (25) clearly visualized membranous structures at high contrast, not only for eukaryotic cells but also for prokaryotes (Fig. 6). This could be exploited to study the symbiotic bacteria that are necessary to establish natural immunity, and also commensal bacteria, including *Helicobactor pylori*.

Today, most kidney biopsies are examined using a combination of three microscopies: OM, immunofluorescence (IF) microscopy and electron microscopy (EM). EM is especially necessary for the diagnosis of the primary glomerulopathies: thin glomerular basement membrane disease, Alport syndrome, fibrillary and/or immunotactoid glomerulonephritis and minimal change disease (30). The diagnosis is basically performed by Epon thin-section TEM (31), and takes a relatively long time due to the required embedding and ultra-thin sectioning procedure. By contrast, ASEM of kidney tissue can be completed within 30 min, or less if the quick UA staining method presented here is employed. With further improvement of the method, ASEM could be an attractive tool for the diagnosis of kidney diseases or to obtain a second opinion. The size of renal biopsies that can be taken from patients is limited. A tissue cube as small as (1×1×5 mm) can be imaged by embedding and TEM, and deliver meaningful results. The open ASEM dish configuration in combination with OM, allows tissue to be manually moved across the flat SiN window using tweezers (Fig. 10). In this way, biopsies of similar size could be investigated by ASEM. As demonstrated with spinal cord tissue, the observation efficiency can also be increased by the use of a new multi-windowed ASEM dish that has an imaging field eight times that of the previous 250×250 μm single-windowed version (Fig. 10). Imaging large areas will be accelerated even more in the future by increasing the number of windows, optimizing their shape and arrangement, and automating sample shifts by constructing motor-driven auto-manipulator over the dish. Two lines of windows with one displaced to give the region a checkered appearance, or a single long narrow rectangular window, would realize the efficient monitoring of a large square area when combined with orthogonal sample shift increments.

During intraoperative cancer diagnosis, the sizes of cell nuclei are usually determined by cryo-sectioning tissue excisons, staining them with H&E and observing them by OM. However, this method takes at least 15–30 min, during which time the tumor excision surgery may have to be suspended. Among the procedures involved in the diagnosis, cryo-thin sectioning is the most difficult, and takes most of the time. Because of the difficulty of this step diagnosis is sometimes confirmed later by paraffin-thin sectioning or occasionally by antibody labeling, involving the possibility of further surgery. When stained with PTA or Pt-blue, nuclei in various tissues, including metastasized cancer cells, were clearly imaged by ASEM without the need for thin-sectioning (Figs. 8 and 9). ASEM observes a 2- to 3-μm specimen layer at 30 kV (12,13,26), which is comparable to the thickness of the cryo-thin sections used for intraoperative diagnosis. Equipped with a fail-safe system against film-window breakage (29), the ASEM has the potential to make diagnoses both quicker and easier, and may be a suitable way to obtain a second opinion. Larger tissue areas could be inspected by moving the sample and by the use of multi-windowed ASEM dishes, as detailed above. To aid diagnosis, the colored OM images of H&E-stained tissue sections more familiar to pathologists should be mimicked by artificially coloring nuclei and cytoplasm in ASEM images blue and red using image recognition algorithms. Furthermore, the immunohistological identification of cancer cells should also be accelerated by the use of immuno-gold labeling and ASEM, as successfully performed for cells cultured on an ASEM dish (12,14,15). In addition, the cathodoluminescence (CL) of luminescent probes is a further option that could be exploited to obtain multi-colored high-resolution images by ASEM (29).

In conclusion, in the present study, we stained different mouse tissues with various heavy metals and imaged them immersed in an open aqueous solution at atmospheric pressure using the ASEM. Not only intercellular connections but also cell organelles were clearly observed in tissues at high resolution. Staining with UA could be achieved in as little as 15 min. After further development of both the staining procedure and the ASEM dish to allow the quick observation of a wider area, this method is expected to be used for rapid intraoperative cancer diagnosis and for the diagnosis of kidney diseases. Furthermore, ASEM can be applied to study the host-microbe interaction that is critical for carcinogenesis in the stomach and also for the body’s homeostasis, especially for the establishment of our natural immunity.

The results presented clearly demonstrate that ASEM is widely applicable to study the basic biology of cells and tissues, to zymology and to food science and industry in general and, perhaps most importantly, to pathology, especially for the diagnosis of cancer and the detection of pathogens during surgery and treatment.

## Abbreviations

ASEM: atmospheric SEM
DDW: double distilled water
EM: electron microscope/microscopy
GA: glutaraldehyde
H&E: hematoxylin and eosin
NCMIR: National Center for Microscopy and Imaging Research
OM: optical microscope/microscopy
PB: phosphate buffer
PBS: phosphate-buffered saline
PFA: paraformaldehyde
PTA: phosphotungstic acid
Pt-blue: platinum-blue, Pt_4_(NH_3_)_8_(C_6_H_13_O_5_)_4_, also known as TI-blue
RT: room temperature
SEM: scanning electron microscope/microscopy
SiN: silicon nitride
TEM: transmission electron microscope/microscopy
THC: tetrahydrocannabinol
UA: uranyl acetate
